# Lateral habenula regulation of emotional hyperthermia: mediation via the medullary raphé

**DOI:** 10.1038/s41598-017-04173-y

**Published:** 2017-06-22

**Authors:** Youichirou Ootsuka, Mazher Mohammed, William W. Blessing

**Affiliations:** Centre for Neuroscience, Department of Human Physiology, School of Medicine, Flinders University, Adelaide, South Australia Australia

## Abstract

The lateral habenula (LHb) has an important role in the behavioural response to salient, usually aversive, events. We previously demonstrated that activation of neurons in the LHb increases brown adipose tissue (BAT) thermogenesis and constricts the cutaneous vascular bed, indicating that the LHb contributes to the central control of sympathetic outflow to thermoregulatory effector organs. We have now investigated whether the LHb mediates BAT thermogenesis elicited by emotional stress, and whether the LHb modulates thermoregulatory sympathetic outflow via the rostral medullary raphé, a key integrative lower brainstem sympathetic control centre. In conscious animals, lesioning the LHb attenuated emotional BAT thermogenesis, suggesting that the LHb is part of the central circuitry mediating emotional hyperthermia. In anesthetized animals, inhibition of neurons in the rostral medullary raphé reversed BAT thermogenesis and cutaneous vasoconstriction elicited by activation of neurons in the LHb, indicating that the LHb-induced autonomic responses are mediated through activation of the rostral medullary raphé neurons. The latency to activate BAT sympathetic discharge from electrical stimulation of the LHb was substantially greater than the corresponding latency after stimulation of the medullary raphé, suggesting that the neuronal pathway connecting those two nuclei is quite indirect.

## Introduction

The lateral habenula (LHb), an evolutionarily-ancient nucleus in the dorsal diencephalon, connects the basal forebrain with midbrain neurons that synthesize dopamine and serotonin^1, 2^. We recently reported that activation of LHb neurons with electrical stimulation and focal injections of bicuculline substantially increases sympathetic outflow to brown adipose tissue (BAT) and to the tail cutaneous vascular bed in anesthetised rats^3^. The increase in heat production by BAT and reduced heat loss via the tail causes an increase in body temperature^4^.

The LHb plays an important role in the behavioural response to adverse, negatively salient environmental situations^1, 5–8^. Physiological responses to the same situations include an increase in body temperature, referred to as emotional hyperthermia or stress-induced hyperthermia, mediated by a combination of BAT thermogenesis and tail artery vasoconstriction, similar to the response obtained by stimulation of the LHb in anesthetized animals^4, 9, 10^. This suggests that the LHb may form part of the brain circuitry mediating emotional hyperthermia.

Currently, there is no information as to whether functional inactivation of the LHb reduces emotional hyperthermia. Nor is there any information concerning the central neural pathways mediating the LHb-induced increases in thermoregulatory sympathetic discharge. The rostral portion of the medullary raphé is a key integrative lower brainstem centre with direct excitatory inputs to the spinal sympathetic neurons that regulate BAT thermogenesis and cutaneous vasoconstriction^11–13^. It is therefore possible that the medullary raphé neurons mediate the LHb-elicited autonomic physiological responses.

In the present study, we first determined, in conscious freely-moving animals, whether electrolytic lesions of the LHb reduce the BAT thermogenesis that contributes to emotional hyperthermia occurring in a rat suddenly confronted with an intruder rat^4, 14^. We then determined, in anesthetized animals, whether inhibition of neurons in the rostral medullary raphé with local injections of muscimol abolishes the LHb-elicited increases in BAT sympathetic discharge, BAT thermogenesis and tail artery constriction. In addition, to provide information concerning the central neural pathways whereby the LHb controls thermoregulatory sympathetic discharge, we used peri-stimulus averaged potential to compare the latencies to activation of BAT sympathetic discharge from the LHb and from the medullary raphe.

## Results

**Figure 1:**
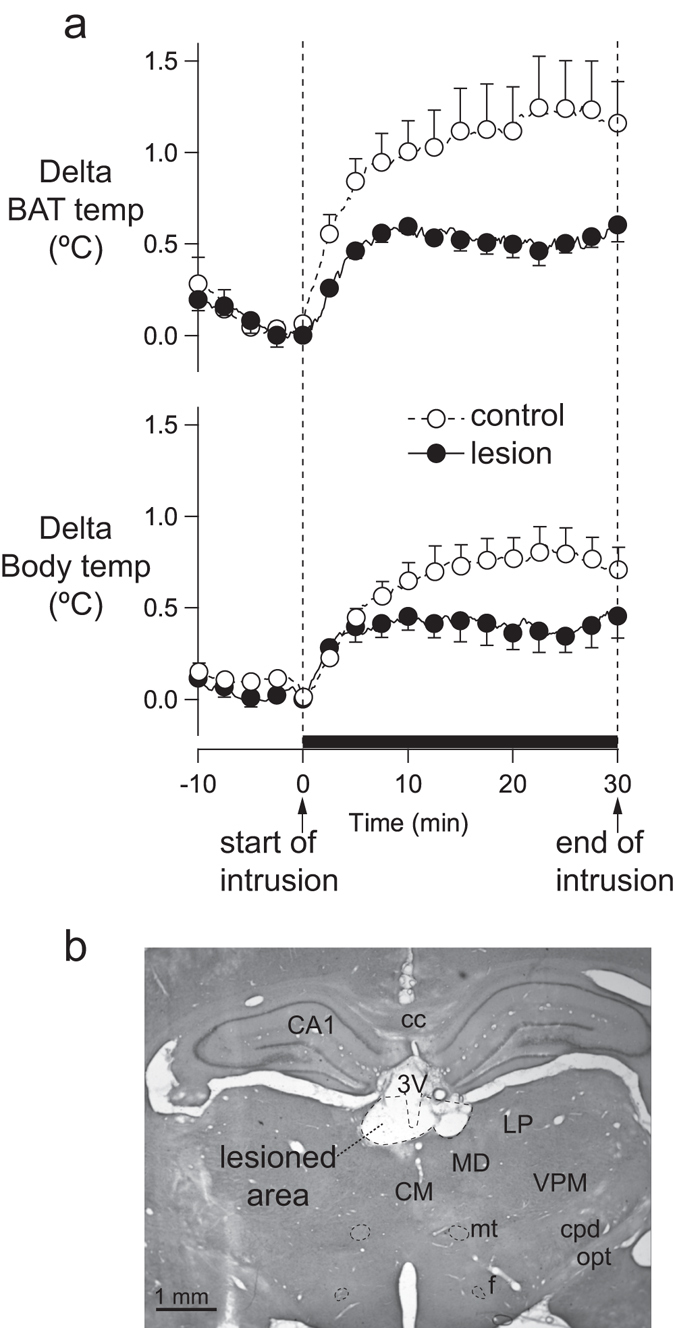
Effect of electrical lesion of the LHb on BAT thermogenesis elicited by the introduction of an intruder in the resident rat cage. (**a**) Averages (mean ± SEM) of original records of BAT and body temperature in the resident rat during the 10 min pre-intruder period and 30 min intruder exposure period. For lesioned rats, data are shown as solid line with filled circled. For control rats, data are shown as dashed lines with open circles. The intrusion period is indicated by the thick horizontal bar. (**b**) Histological demonstration of electrically lesioned area in the LHb. CA1, field CA of hippocampus; cc, corpus callosum; CM, central medial thalamic nucleus; cpd, cerebral peduncle; f, fornix; LP, lateral posterior thalamic nucleus; MD, mediodorsal thalamic nucleus; mt, mammilothalamic tract; opt, optic tract.

In the conscious rat study, after the introduction of an intruder rat to the resident rat cage, BAT and body temperature of the sham-lesioned resident rat increased (n = 5) (Fig. 1a), as previously reported^4^. As is also apparent in Fig. 1a, in the LHb-lesioned rats (n = 5), the amplitudes of the increase in BAT and body temperature were substantially reduced (BAT temperature, 0.6 ± 0.1 °C vs 1.2 ± 0.2 °C, P = 0.028, unpaired t-test; body temperature, 0.5 ± 1 °C vs 0.8 ± 0.1 °C, P = 0.042, unpaired t-test). An example of the histological appearance of the LHb in electrically lesioned rats is shown in Fig. 1b.

**Figure 2:**
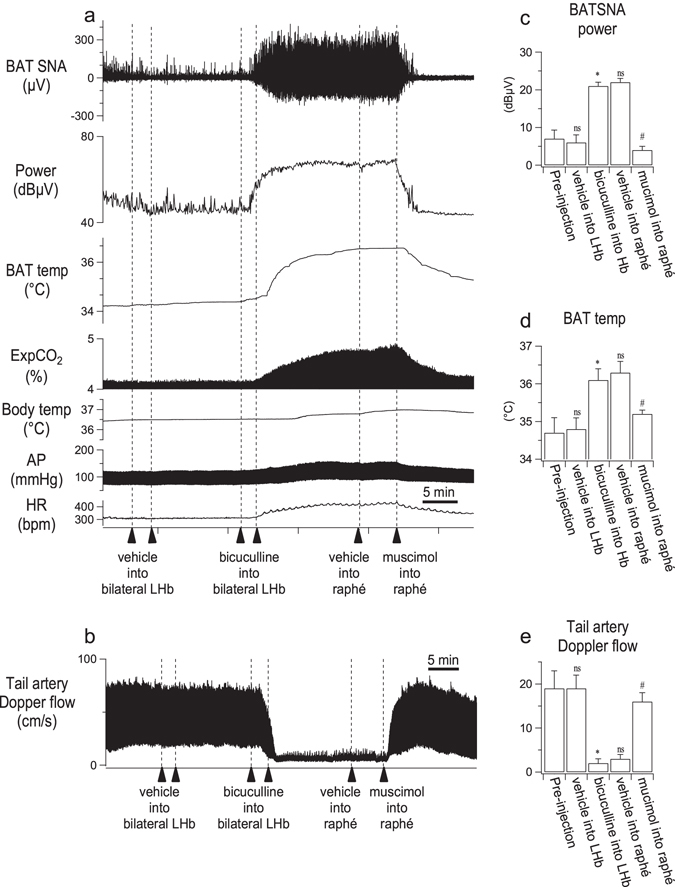
Effect of muscimol injection (0.5 nmol in 50 nl) into the medullary raphé on autonomic response elicited by bilateral injections of bicuculline (1 nmol in 100 nl) into the LHb. (**a**) Chart records of simultaneously recorded brown adipose tissue sympathetic nerve discharge (BAT SNA), its spectral power, BAT temperature (BAT temp), end expiratory CO_2_ (ExpCO_2_), body temperature (Body temp), arterial pressure (AP) and heart rate (HR). (**b** and **c**) Group data (mean ± SEM) for mean effects of vehicle/drug on BAT SNA power and BAT temperature. (**d**) Chart record of tail artery Doppler flow signal. (**e**) Group data (mean ± SEM) for mean effects of vehicle/drug on tail artery Doppler flow. In **a** and **d**, sequential experimental manipulations of LHb and medullary raphé function are indicated by the vertical arrows. ‘ns’ not significantly different from the preceding injection level (pre-injection or bicuculline into LHb), P > 0.05; *significant different from the pre-injection value, *P* < 0.001; ^#^significant different from the post-bicuculline value, *P* < 0.001.

In the anesthetized rat study (n = 6), bilateral injections into the LHb of bicuculline (1 nmol in 100 nl) and then muscimol (0.5 nmol in 50 nl) into the medullary raphé, but not vehicle, significantly altered BAT sympathetic nerve discharge (*F*_*4,20*_ = 68.514*, P* < 0.001, repeated measures ANOVA), as can be seen in Fig. 2a,c. Bicuculline increased BAT sympathetic nerve discharge from 6 ± 2 to 21 ± 1 dBµV (n = 6, P = 0.001, Fisher’s LSD) within 10 min and subsequent injection of muscimol into the medullary raphé decreased the discharge from 22 ± 1 to 4 ± 1 dBµV (n = 6, P = 0.001, Fisher’s LSD), a value similar to the pre-bicuculline value. The vehicle injections into LHb and the medullary raphé did not affect discharge (n = 6, P > 0.1 for all post-hoc comparisons, Fisher’s LSD).

Similar results were obtained for BAT and body temperatures (BAT temperature *F*_*4,20*_ *= *56.514*, P* < 0.001, repeated measures ANOVA; body temperature *F*_*4,20*_ = 7.668*, P* = 0.001, repeated measured ANOVA). Bicuculline increased BAT temperature from 34.8 ± 0.3 to 36.1 ± 0.3 °C (n = 6, P < 0.001, Fisher’s LSD) and body temperature from 36.8 ± 0.2 to 37.1 ± 0.2 °C (n = 6, P = 0.006, Fisher’s LSD). Subsequent injection of muscimol into the medullary raphé decreased BAT temperature from 36.3 ± 0.3 °C to 35.3 ± 0.3 °C (n = 6, P < 0.001, Fisher’s LSD) and body temperature from 37.2 ± 0.2 to 36.7 ± 0.2 °C (n = 6, P = 0.02, Fisher’s LSD). The vehicle injections into LHb and the medullary raphé did not affect BAT or body temperature (P > 0.08 for all post-hoc comparisons, Fisher’s LSD). End expiratory CO_2_ also showed a similar pattern (*F*_*4,20*_ *= *31.941*, P* < 0.001, repeated measures ANOVA). Bicuculline increased CO_2_ from 4.7 ± 0.2 to 5.4 ± 0.2% (n = 6, P = 0.001, Fisher’s LSD). Subsequent injection of muscimol into the medullary raphé decreased CO_2_ from 5.5 ± 0.2 to 4.8 ± 0.2% (n = 6, P < 0.001, Fisher’s LSD). Vehicle injections into LHb and the medullary raphé did not affect CO_2_ (n = 6, P > 0.3, for all post-hoc comparisons, Fisher’s LSD).

Arterial blood pressure and heart rate were also significantly changed by injection of bicuculline into the LHb and subsequent injection of muscimol into the medullary raphé (arterial pressure, *F*_*4,20*_ *= *97.141*, P* < 0.001, repeated measures ANOVA; heart rate *F*_*4,20*_ = 11.342*, P* < 0.001, repeated measures ANOVA). Bicuculline increased arterial pressure from 107 ± 4 to 132 ± 4 mmHg (n = 6, P < 0.001, Fisher’s LSD) and heart rate from 338 ± 11 to 422 ± 7 bpm (n = 6, P < 0.001, Fisher’s LSD). Subsequent injection of muscimol into the medullary raphé reversed these changes. The muscimol injection decreased arterial pressure from 132 ± 4 to 117 ± 4 mmHg (n = 6, P < 0.001, Fisher’s LSD) and heart rate from 416 ± 15 to 353 ± 28 bpm (n = 6, P = 0.034, Fisher’s LSD). Vehicle injections in the LHb and the medullary raphé did not affect arterial pressure or heart rate (n = 6, P > 0.4 for all post-hoc comparisons, Fisher’s LSD).

In the blood flow-recording group (n = 7), tail artery blood flow Doppler signal was markedly affected by injection of bicuculline into the LHb and subsequent injection of muscimol into the medullary raphé (*F*_*4,24*_ = 22.840*, P* < 0.001, repeated measures ANOVA) (Fig. 2d and e). Bicuculline reduced the flow signal to 13 ± 3% of pre-injection level (19 ± 2 to 2 ± 1 cm/s, n = 7, P = 0.002, Fisher’s LSD). Subsequent injection of muscimol into the medullary raphé increased to flow signal to 94 ± 15% of the pre-bicuculline level (from 3 ± 1 to 16 ± 2 cm/s, n = 7, P < 0.001, Fisher’s LSD). Vehicle injections into the medullary raphé caused a slight increase in the tail artery blood flow (from 2 ± 1 to 3 ± 1 cm/s, n = 7, P = 0.015, Fisher’s LSD). Vehicle injection into the LHb did not affect the tail artery blood flow signal (n = 7, P = 0.443, Fisher’s LSD).

**Figure 3:**
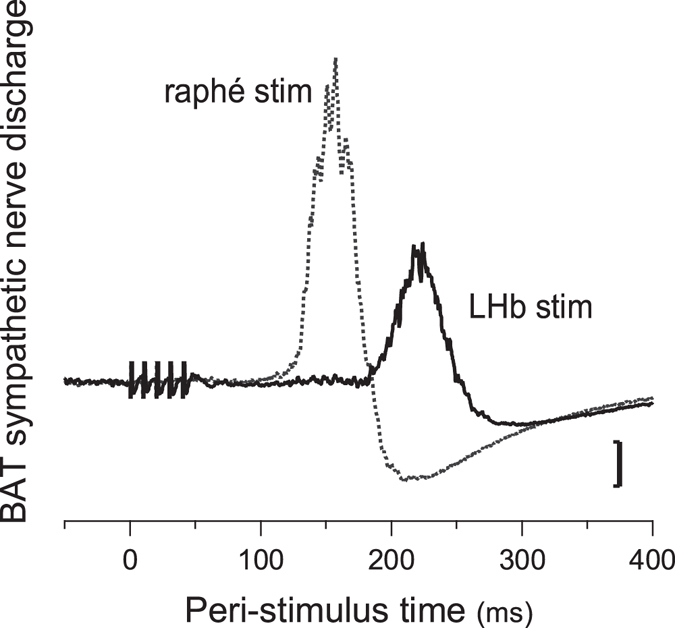
Peri-stimulus averaged potentials in BAT sympathetic nerve evoked by pulsed electrical stimulation of the LHb or the medullary raphé.

In the BAT nerve-recording group, the LHb and the medullary raphé were also electrically stimulated in 5 of the 7 animals. Stimulation of LHb evoked discharge of the BAT sympathetic nerve with an onset latency of 148 ± 3ms and a peak latency of 209 ± 6 ms (n = 5) (Fig. 3). Stimulation of the rostral medullary raphé evoked discharge of the BAT sympathetic nerve with an onset latency of 115 ± 5 ms and a peak latency of 160 ± 8 ms (n = 5). These two latencies were significantly different (P = 0.03, paired t-test), and the difference between these two latencies was 33 ± 4 ms with 7.9 ± 0.5 mm distance.

**Figure 4:**
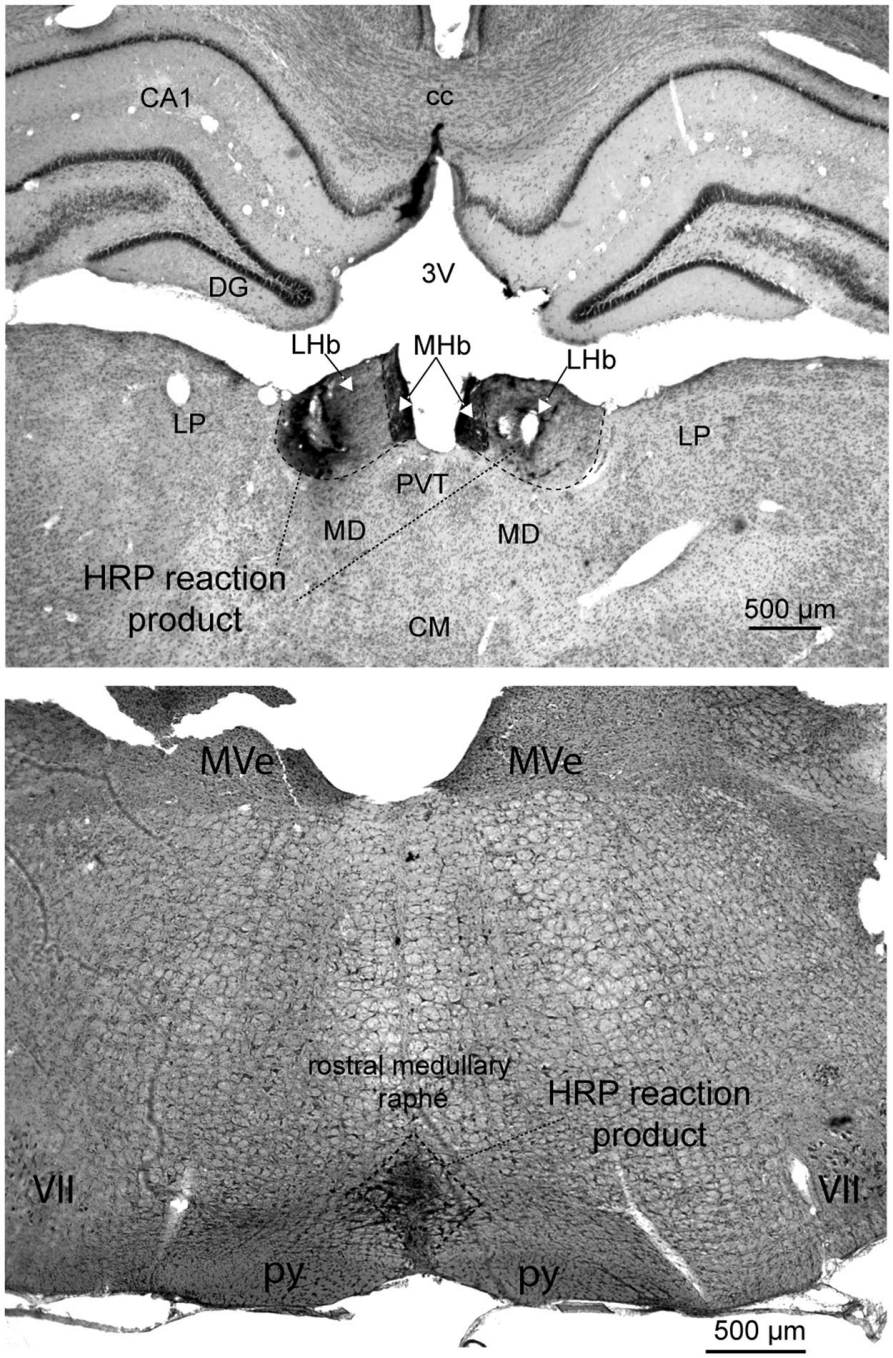
Histological demonstration of microinjection sites into the LHb (top) or into the medullary raphé (bottom). The sites were marked by horseradish peroxidase (HRP) reaction product. *Top*: CA1, field CA of hippocampus; cc, corpus callosum; CM, central medial thalamic nucleus; DG, dentate gyrus; LHb, lateral habenula; LP, lateral posterior thalamic nucleus; MD, mediodorsal thalamic nucleus; MHb, medial habenula; PVT, paraventricular thalamic nucleus. *Bottom:* MVe, medial vestibular nucleus; py, pyramidal tract; VII, facial nucleus.

An example of LHb and the medullary raphé injections sites marked by horseradish peroxidase reaction product is shown in Fig. 4.

## Discussion

The LHb, a link between forebrain networks and midbrain monoaminergic nuclei, plays an important role in the individual’s corrective behavioural response to unfavourable environmental occurrences, both in experimental animals and in humans^5, 7, 8^. Our recent report demonstrated, for the first time, that activation of the LHb also engages brain control of the autonomic functions that are integrated with the corrective behavioural responses^3^. The LHb-elicited increase in BAT thermogenesis contributed to a rise in body temperature. Our present result in conscious unrestrained rats shows that prior destruction of the LHb reduces the intensity of the rise in body temperature elicited by a salient, threatening, environmental situation, and that reduced BAT thermogenesis contributes to this reduced emotional hyperthermia.

We also show, for the first time, that chemical activation of the LHb increases discharge in BAT sympathetic nerves, confirming that the LHb-elicited BAT thermogenesis is sympathetically mediated. The medullary raphé is established as the lower brainstem centre directly controlling spinal sympathetic neurons that regulate BAT thermogenesis and cutaneous vasoconstriction^11–13, 15^. Our study firmly establishes that inhibition of neurons in the medullary raphé reverses BAT thermogenesis and cutaneous vasoconstriction elicited by activation of neurons in the LHb. Thus the medullary raphé is also likely to relay LHb-mediated emotional BAT thermogenesis and cutaneous vasoconstriction. This is consistent with previous studies demonstrating that inactivation of neurons in the medullary raphé abolishes emotionally-induced BAT thermogenesis and cutaneous vasoconstriction^16, 17^.

The major LHb direct efferent projection is via the fasciculus retroflexus to midbrain and pontine regions including the midbrain and pontine raphé nuclei, the rostromedial tegmental nucleus (RMTg) and the ventral tegmental area (VTA)^2, 18–20^. The corrective behavioural response is mediated via an excitatory drive from the LHb to GABAergic neurons in the RMTg. Their direct inhibitory projection to dopamine-synthesizing neurons in the VTA constitutes the “dopamine brake”, an action that reduces the activity of the rewarding mesolimbic dopaminergic projection from the VTA to forebrain centres including the nucleus accumbens and the medial prefrontal cortex^21^. A recent study supports this idea by demonstrating that lesioning the RMTg releases the dopamine brake driven by the LHb^22^. These dopamine-related nuclei may be also involved in physiological autonomic responses associated with the corrective behavioural response (see further discussion in below).

Our present study establishes that final component of the pathway involves the medullary raphé, the region that functions as the lower brainstem centre controlling spinal sympathetic outflow to BAT^12^. The neural projection mediating the LHb-controlled increase in BAT thermogenesis must therefore be via the medullary raphé. However the precise anatomical connections have not been determined. Anatomical tracing studies show that there are no direct monosynaptic projections from the LHb to the medullary raphé^23, 24^. The mediating neuroanatomical pathway must therefore be indirect and multisynaptic. The latency findings of our present study provide strong evidence supporting this conclusion. The onset latency of BAT nerve discharges evoked by electrical simulation in the LHb is 148 ms, more than 30 ms greater than the corresponding latency (115 ms) following stimulation of the medullary raphé.

Indeed, the projection from the LHb to the medullary raphé must be quite indirect because the 30 ms latency difference between the LHb and the medullary raphé is greater than 25 ms corresponding latency difference between the medullary raphé and the preoptic area, a thermoregulatory centre more rostral than the LHb^25^. This raises the possibility that the thermoregulatory actions of the LHb might also be mediated via the dopamine braking action of the RMTg on the VTA neurons, implying that activity of VTA dopamine neurons normally inhibits BAT thermogenesis. There is evidence supporting this hypothesis. Inhibition of the VTA region increases BAT temperature^26^. Apomorphine, a drug that acts by stimulating dopamine D2 receptors, has long been known to reduce body temperature^27–29^ and our laboratory has demonstrated that the hypothermic action of dopamine D2 receptor-stimulating agents results from inhibition of sympathetic outflow to BAT and to the tail vasoconstrictor bed^30, 31^. It is thus of major interest that direct injection of apomorphine or dopamine itself into the nucleus accumbens, a major target of the VTA dopamine neurons, substantially lowers body temperature^32^. The medial prefrontal cortex is another target of the VTA^33^ and preliminary results also suggest an important role for this region in controlling BAT thermogenesis in response to stress^9^. Thus the LHb, via the dopamine brake, may contribute to the emotional hyperthermia that results from salient negative experiences^4^. Since there are no direct connection from the VTA to the medullary raphé^24, 34, 35^, the dopamine brake signal pathway via VTA to the medullary raphé must be also indirect.

## Methods

All experiments were performed on male Sprague-Dawley rats (300–400 g) with procedures approved by the Animal Welfare Committee of Flinders University. The methods were carried out in accordance with the Australian code for the care and use of animals for scientific purposes 8^th^ edition.

### Conscious animal experiments

For preparatory surgery, rats (n = 10) were anaesthetized with 2% isoflurane. Burr-hole craniotomy was performed for electrical lesion of the LHb. The left and right LHb was lesioned by passing DC current (0.5–1 mA, 20 sec) via a glass-coated tungsten electrode (n = 5). Sham operation was made in the control group (n = 5). Pre-calibrated thermistor probes were chronically implanted in the interscapular BAT (BAT temperature) and in the mediastinum ventral to the trachea (body temperature)^36^. Insulated wired from the temperature probes passed subcutaneously and connected to a head socket. Then the socket was attached to the skull with screws and dental cement^36^. At the end of the preparatory surgery, antibiotic (enrofloxacin, 5 mg/kg s.c.) and analgesic (carprofen, 5 mg/kg s.c.) were administered. After the surgery, each rat was individually housed for at least 1-week recovery period under a reverse light-dark cycle (lights off at 0700 h and light on at 1900 h).

At least 12 hours before experiments, the rat was transferred to an experimental room and placed in a recording cage in a temperature-controlled chamber. The ambient temperature of the chamber was maintained at 24–26 °C. The resident rat’s head socket was connected to recording devices via a flexible cable and a counter-balanced swivel device (SL12C, PlasticsOne). Temperature signals were amplified with a manually-made bridge amplifier. During recording, the resident rat could access food and water ad libitum. The weight of the food container was continuously measured with a strain gauge, so that the timing of eating and the amount of food eaten was measured^37^. The intruder rat confined to a small cage was introduced to the cage of the resident rat during the dark phase approximately 30 min after the end of a meal when BAT and body were at low levels^4, 14^. The intruder was removed 30 min after the introduction.

### Anaesthetised animal experiments

BAT sympathetic nerve discharge, together other autonomic parameters, was recorded in one group of animals (n = 6). Tail blood flow Doppler signal, together with other autonomic parameters, was recorded in a separate group of rats (n = 7). In this group of animals, a blood flow probe was implanted under 2% isoflurane^4, 38^ at least a week before experiments. At the end of the preparatory surgery, antibiotic (enrofloxacin, 5 mg/kg s.c.) and analgesic (carprofen, 5 mg/kg s.c.) were administered.

On the day of experiment, rats were anesthetized with isoflurane (2% in oxygen), and an endotracheal tube was inserted via tracheotomy. The right femoral vein and artery were cannulated for injection of drug and measurement of arterial pressure, respectively. The level of anaesthesia was maintained at a depth sufficient to abolish paw withdrawal reflexes. Rats were then mounted in prone position in a stereotaxic setup. Burr-hole craniotomy was performed to allow for chemical injection into left and right LHb (5.2–5.4 rostral from the interaural line, 0.6–0.7 mm lateral from midline, and 4.4–4.6 mm deep from cortex surface), and into the rostral medullary raphé (3.0 caudal from the interaural line, midline, 9.2 mm deep from cortex surface). The rats were then paralyzed with d-tubocurarine (initially 0.3 mg i.v. (1–1.6 mg/kg), thereafter, 0.3 mg i.v. every 1–1.5 h) and ventilated artificially with 100% O_2_ (60–65 cycle/min, 2–3 ml/cycle). The animal was allowed to recover from paralysis between doses so that adequate anaesthesia could be confirmed before paralysis was re-established. In the BAT sympathetic nerve recording group, the interscapular BAT sympathetic nerve was isolated as described previously^39^, and covered with paraffin oil to prevent drying.

End expiratory CO_2_ (ExpCO_2_) was maintained at 4–5% at resting condition. BAT, rectal (body) and abdominal skin temperatures were measured with thermocouples (TC-2000; Sable Systems). Body temperature was maintained at 35–36 °C with a water jacket^40^. Arterial pressure was measured using a transducer and a bridge amplifier. An electrocardiogram (ECG) was amplified and filtered (x5,000, band-pass 70–5000 Hz, NT114, Digitimer Ltd.). Nerve activity was recorded with a pair of silver wire electrodes, amplified (x20,000, NL104, Digitimer Ltd.) and filtered (band-pass 1–1000 Hz, NL125, Digitimer Ltd.). Tail artery Doppler blood flow signals were monitored using System 6 Model (Triton Technology).

In the BAT recording group, the skin of the body trunk was cooled by perfusing cold water (5–10 °C) through the water jacket for 2–3 min to confirm that recording was made from sympathetic nerves innervating BAT^39^. Only animals in which the cooling increased BAT nerve discharge were used in experiments.

We obtained peri-stimulus averaged potentials in BAT sympathetic discharge elicited by electrical stimulation of LHb (5 pulses, 0.5 ms duration, 10 ms interval, 500 µA-1.2 mA) and of the medullary raphé (3 pulses, 0.5 ms duration, 10 ms interval, 50–100 µA). At the end of experiments, ganglionic blockade with chlorisondamine chloride (10 mg/kg, i.v.) was administered to confirm that the absence of BAT sympathetic nerve activity elicited by electrical stimulation of the rostral medullary raphé to verify that nerve recording was from postganglionic sympathetic axons.

A glass micropipette filled with either vehicle (water) or bicuculline ((−)-bicuculline methiodide in vehicle) was inserted into the LHb. The water vehicle (100 nl) was firstly injected, and the response recorded. After at least 10 min, 100 nl of bicuculline (1 nmol) was injected into LHb bilaterally. Ten to fifteen minutes later when BAT sympathetic was increased or tail cutaneous blood flow reduced, water vehicle (50 nl) was injected into the medullary raphé. After at least 5 min, 50 nl of muscimol (0.5 nmol) was injected into the rostral medullary raphé. At least 5 min observation time was taken after each injection even if no response was observed.

### Histology

At the end of experiment, the rats were deeply anesthetized with pentobarbitone sodium (over 80 mg/kg i.v.) and perfused transcardially with washing solution (140 mM NaNO_2_, 1 mM phosphate buffer pH 7.4; 0.2 mM NaH_2_PO_4_.H_2_O, 0.8 mM NaH_2_PO_4_) followed by formaldehyde-fixatives (3.6% formaldehyde stabilized 1% methanol in 10 mM phosphate buffer). The brain was removed for histological confirmation of injection site by visualization of horseradish reaction products. Horseradish peroxidase (Peroxidase from horseradish Type VI) was added to the drug injectate. For the horseradish peroxidase reaction, the brain sections were incubated in 0.05% diaminobenzidine solution (3,3′-Diaminobenzidine in 0.1 M Tris buffer saline pH 7.4; 0.1 M Trizma base in saline) for 10 min. Hydrogen peroxide was added to the incubation solution (0.37 µl of 30% H_2_O_2_ per 1 ml of the diaminobenzidine solution)^3^.

### Data analysis

In the anaesthetized animal experiments, all data were sampled and digitized by PowerLab (ADInstruments Inc.) at 1 kHz. Heart rate was computed from ECG. Data were analysed with Igor Pro (WaveMetrics Inc.). The amplitude of BAT sympathetic nerve discharges was expressed as total power spectral density between 0 and 20 Hz from the autospectra of sequential 5.12-s segments of BAT sympathetic nerve activity^40, 41^. The total power was shown in decibel microvolt (10 × log (power), dBµV). In the conscious animal studies, all data are digitized at 1 Hz with the PowerLab. Post-intruder values averaged the signals from 18 to 28 min after introduction of the intruder rat. Statistical analysis was performed using SPSS (IBM Corp.). Group data were shown as mean ± SEM. For the conscious rat studies, control and lesioned animals were compared using an unpaired t-test (two-sided) for independent samples. Results from the anaesthetized rat study were analysed by repeated measures ANOVA. Fisher’s LSD was used as a post hoc test. Onset latencies of BAT nerve discharge elicited by electrical stimulation into the LHb and the medullary raphé were analysed by a paired t-test (two-sided). The limit for statistical significance was set at the *P* = 0.05 level.

## References

[1] Hikosaka O (2010). The habenula: from stress evasion to value-based decision-making. Nat Rev Neurosci.

[2] Quina LA (2014). Efferent Pathways of the Mouse Lateral Habenula. J. Comp. Neurol..

[3] Ootsuka Y, Mohammed M (2015). Activation of the habenula complex evokes autonomic physiological responses similar to those associated with emotional stress. Physiological Reports.

[4] Mohammed M, Ootsuka Y, Blessing W (2014). Brown adipose tissue thermogenesis contributes to emotional hyperthermia in a resident rat suddenly confronted with an intruder rat. Am. J. Physiol. Regul. Integr. Comp. Physiol.

[5] Matsumoto M, Hikosaka O (2007). Lateral habenula as a source of negative reward signals in dopamine neurons. Nature.

[6] Matsumoto M, Hikosaka O (2009). Representation of negative motivational value in the primate lateral habenula. Nat. Neurosci..

[7] Stamatakis AM, Stuber GD (2012). Activation of lateral habenula inputs to the ventral midbrain promotes behavioral avoidance. Nat. Neurosci..

[8] Lawson RP (2014). The habenula encodes negative motivational value associated with primary punishment in humans. Proc. Natl. Acad. Sci. USA.

[9] Kataoka, N. & Nakamura, K. Direct pathway from ventral medial prefrontal cortex to dorsomedial hypothalamus drives psychological stress-induced hyperthermia. *The FASEB Journal* **30**, 994.992 (2016).

[10] Nakamura K (2015). Neural circuit for psychological stress-induced hyperthermia. Temperature.

[11] Blessing W, McAllen R, McKinley M (2016). Control of the Cutaneous Circulation by the Central Nervous System. Comprehensive Physiology.

[12] Morrison SF, Madden CJ (2014). Central nervous system regulation of brown adipose tissue. Comprehensive Physiology.

[13] Ootsuka Y, Tanaka M (2015). Control of cutaneous blood flow by central nervous system. Temperature.

[14] Mohammed M, Ootsuka Y, Yanagisawa M, Blessing W (2014). Reduced brown adipose tissue thermogenesis during environmental interactions in transgenic rats with ataxin-3-mediated ablation of hypothalamic orexin neurons. Am. J. Physiol. Regul. Integr. Comp. Physiol..

[15] Morrison SF, Madden CJ, Tupone D (2014). Central neural regulation of brown adipose tissue thermogenesis and energy expenditure. Cell metabolism.

[16] Kataoka, N., Hioki, H., Kaneko, T. & Nakamura, K. Psychological Stress Activates a Dorsomedial Hypothalamus-Medullary Raphe Circuit Driving Brown Adipose Tissue Thermogenesis and Hyperthermia. *Cell metabolism*, doi:10.1016/j.cmet.2014.05.018 (2014). 10.1016/j.cmet.2014.05.018 24981837

[17] Ootsuka Y, Blessing WW (2005). Inhibition of medullary raphe/parapyramidal neurons prevents cutaneous vasoconstriction elicited by alerting stimuli and by cold exposure in conscious rabbits. Brain Res..

[18] Herkenham M, Nauta WJ (1979). Efferent connections of the habenular nuclei in the rat. J. Comp. Neurol..

[19] Sutherland RJ (1982). The dorsal diencephalic conduction system: a review of the anatomy and functions of the habenular complex. Neurosci. Biobehav. Rev..

[20] Yetnikoff, L., Cheng, A. Y., Lavezzi, H. N., Parsley, K. P. & Zahm, D. S. Sources of input to the rostromedial tegmental nucleus, ventral tegmental area, and lateral habenula compared: A study in rat. *J. Comp. Neurol*., doi:10.1002/cne.23797 (2015). 10.1002/cne.23797 PMC4575621 25940654

[21] Bourdy R, Barrot M (2012). A new control center for dopaminergic systems: pulling the VTA by the tail. Trends Neurosci..

[22] Brown PL (2017). Habenula-Induced Inhibition of Midbrain Dopamine Neurons Is Diminished by Lesions of the Rostromedial Tegmental Nucleus. J. Neurosci..

[23] Araki M, McGeer PL, Kimura H (1988). The efferent projections of the rat lateral habenular nucleus revealed by the PHA-L anterograde tracing method. Brain Res..

[24] Hermann DM, Luppi PH, Peyron C, Hinckel P, Jouvet M (1997). Afferent projections to the rat nuclei raphe magnus, raphe pallidus and reticularis gigantocellularis pars alpha demonstrated by iontophoretic application of choleratoxin (subunit b). J. Chem. Neuroanat..

[25] Tanaka M, McKinley MJ, McAllen RM (2011). Preoptic-Raphe Connections for Thermoregulatory Vasomotor Control. J. Neurosci..

[26] Shibata M (1996). Procaine microinjection into the lower midbrain increases brown fat and body temperatures in anesthetized rats. Brain Res..

[27] Cox B, Kerwin R, Lee TF (1978). Dopamine receptors in the central thermoregulatory pathways of the rat. J Physiol.

[28] Chipkin RE (1988). Effects of D1 and D2 antagonists on basal and apomorphine decreased body temperature in mice and rats. Pharmacol. Biochem. Behav..

[29] Ogren SO, Fuxe K (1988). Apomorphine and pergolide induce hypothermia by stimulation of dopamine D-2 receptors. Acta Physiol. Scand..

[30] Blessing WW, Ootsuka Y (2007). Activation of dopamine D2 receptors in the CNS inhibits sympathetic cutaneous vasomotor alerting responses (SCVARs), contributing to clozapine’s SCVAR-inhibiting action. Prog. Neuropsychopharmacol. Biol. Psychiatry.

[31] Ootsuka Y, Heidbreder CA, Hagan JJ, Blessing WW (2007). Dopamine D(2) receptor stimulation inhibits cold-initiated thermogenesis in brown adipose tissue in conscious rats. Neuroscience.

[32] Grabowska M, Anden NE (1976). Apomorphine in the rat nucleus accumbens: effects on the synthesis of 5-hydroxytryptamine and noradrenaline, the motor activity and the body temperature. J. Neural Transm..

[33] Morales M, Margolis EB (2017). Ventral tegmental area: cellular heterogeneity, connectivity and behaviour. Nat Rev Neurosci.

[34] Beckstead RM, Domesick VB, Nauta WJ (1979). Efferent connections of the substantia nigra and ventral tegmental area in the rat. Brain Res..

[35] Oades RD, Halliday GM (1987). Ventral tegmental (A10) system: neurobiology. 1. Anatomy and connectivity. Brain Res..

[36] Ootsuka Y (2009). Brown adipose tissue thermogenesis heats brain and body as part of the brain-coordinated ultradian basic rest-activity cycle. Neuroscience.

[37] Blessing W, Mohammed M, Ootsuka Y (2012). Heating and eating: brown adipose tissue thermogenesis precedes food ingestion as part of the ultradian basic rest-activity cycle in rats. Physiol. Behav..

[38] de Menezes RC, Ootsuka Y, Blessing WW (2009). Sympathetic cutaneous vasomotor alerting responses (SCVARs) are associated with hippocampal theta rhythm in non-moving conscious rats. Brain Res..

[39] Ootsuka Y, McAllen RM (2006). Comparison between two rat sympathetic pathways activated in cold defense. Am. J. Physiol. Regul. Integr. Comp. Physiol..

[40] Ootsuka Y, Kulasekara K, de Menezes RC, Blessing WW (2011). SR59230A, a beta-3 adrenoceptor antagonist, inhibits ultradian brown adipose tissue thermogenesis and interrupts associated episodic brain and body heating. Am. J. Physiol. Regul. Integr. Comp. Physiol..

[41] Madden CJ, Morrison SF (2006). Serotonin potentiates sympathetic responses evoked by spinal NMDA. J. Physiol. (Lond)..

